# Long Term Follow-Up of Polish Patients with Isovaleric Aciduria. Clinical and Molecular Delineation of Isovaleric Aciduria

**DOI:** 10.3390/diagnostics10100738

**Published:** 2020-09-23

**Authors:** Edyta Szymańska, Aleksandra Jezela-Stanek, Anna Bogdańska, Dariusz Rokicki, Ewa Ehmke vel Emczyńska-Seliga, Magdalena Pajdowska, Elżbieta Ciara, Anna Tylki-Szymańska

**Affiliations:** 1 Department of Gastroenterology, Hepatology, Feeding Disorders and Pediatrics, Children’s Memorial Health Institute, 01-138 Warsaw, Poland; edyta.szymanska@onet.com.pl; 2 Department of Genetics and Clinical Immunology, National Institute of Tuberculosis and Lung Diseases, 01-138 Warsaw, Poland; jezela@gmail.com; 3 Department of Biochemistry, Radioimmunology and Experimental Medicine, Children’s Memorial Health Institute, 04-730 Warsaw, Poland; a.bogdanska@ipczd.pl (A.B.); m.pajdowska@ipczd.pl (M.P.); 4 Department of Pediatrics, Nutrition and Metabolic Disorders, Children’s Memorial Health Institute, 04-730 Warsaw, Poland; d.rokicki@ipczd.pl (D.R.); e.emczynska@ipczd.pl (E.E.v.E.-S.); 5 Department of Medical Genetics, Children’s Memorial Health Institute, 04-730 Warsaw, Poland; e.ciara@ipczd.pl

**Keywords:** isovaleric acidemia, newborn screening, dietary management, organic acidurias, *IVD* gene, mild phenotype

## Abstract

Isovaleric acidemia (IVA) is an autosomal recessive leucine inborn error of metabolism caused by isovaleryl-CoA dehydrogenase deficiency. The disease has various courses, from severe ones manifesting in newborns to the intermittent form with first manifestation in children and adults. The aim of this study was to analyze clinical and neurological outcomes in Polish patients with IVA. Ten patients diagnosed and treated in The Children’s Memorial Health Institute were included in the study. The diagnosis was based on tandem MS (increased level of C5 acylcarnitine) and urine GCMS (increased isovalerylglycine, and 3-hydroxyisovaleric acid). Molecular analysis was performed in seven patients (70%) leading to the detection of pathogenic variants in the *IVD* gene in all of them. A retrospective analysis of patients’ medical records included: demographics, symptoms at diagnosis, medical management, and biochemical and clinical outcomes following therapy. The median follow-up time (median; Q1–Q2) was 2.5 years (1.5–9.0) for newborn screening (NBS) and family screening (FS) children, and 17 years (5.0–20) for symptomatic patients. Five patients were in a good clinical state, four children presented mild neurological symptoms, and one—severely delayed child. In the *IVD* gene, five known and two novel variants (p.466C>G, c.1132G>A) were identified. Molecular analysis was performed in seven patients leading to identification of biallelic pathogenic variants in the *IVD* gene in all of them. We can conclude that long-term clinical and neurological outcomes of patients with IVA were satisfactory as a result of an early diagnosis and proper management. Although early treatment did not prevent decompensations, they were milder in these patients.

## 1. Introduction

Isovaleric acidemia (IVA, OMIM: 243500) is an autosomal recessive leucine inborn error of metabolism caused by the deficiency of mitochondrial isovaleryl-CoA dehydrogenase (IVD; EC 1.2.99.10), encoded by the *IVD* gene. It is one of the four “classical” organic acidemias: propionic (PA), methylmalonic (MMA), glutaric (GA), and IVA, with a prevalence 1–9/100,000 [1].

The *IVD* gene was mapped on chromosome 15q14-q15 [2]. It encodes a homonymous protein, IVD enzyme, which catalyses the third step in leucine catabolism. Its deficiency results in the accumulation of isovaleryl-CoA, such as free isovaleric acid, 3-hydroxyisovaleric acid (3-HIVA), isovaleryl (C5)-carnitine, and isovalerylglycine (IVG). Isovaleric acid and IVG are potentially neurotoxic [3].

According to the standard classification, two types are distinguished: acute neonatal and chronic intermittent form. Encephalopathy, developmental delay, lethargy, sweaty feet odour, and tremor are among the main characteristics of the neonatal form. Features of the intermittent type of IVA are usually triggered by a stress such as infection, fever, or excessive intake of high-protein foods [4].

Molecular analysis of the *IVD* gene has allowed for the identification of different types of pathogenic variants, with no straightforward phenotype–genotype correlation in most cases. One of them, missense mutation, c.941C>T, p.(Ala314Val) is common in patients identified through newborn screening (NBS) with mild metabolite elevations and those who have remained asymptomatic through following years [5,6].

As far as IVA clinical course is concerned, metabolic decompensations are the most serious complication. Laboratory investigations show metabolic acidosis and ketoacidosis, hyper- or hypoglycemia (especially in neonatal period), hyperglycinemia (inhibition of glycine degradation), hyperammonemia (inhibition of the urea cycle), neutropenia, or pancytopenia (bone marrow suppression) [7,8]. Since acute metabolic decompensation is almost always precipitated by a stressor, it is important to identify and eliminate it [9].

Additionally, carnitine and/or glycine is administered to enhance the conversion of potentially neurotoxic products (free isovaleric acid) into non-toxic carnitine and glycine conjugates which are easily excreted in the urine [1]. It is of importance to mention that different emergency management protocols are applied depending on metabolic stress (e.g., illness and fasting) [1]. Unfortunately, as a result of IVA’s heterogeneity, rarity, and lack of extensive, multi-center, longitudinal studies on patient outcomes, there is disagreement regarding optimal dietary management [10].

In 2014, the web-based E-IMD IVA Guidelines were published, which stated that natural protein intake should be restricted to reduce the isovaleric acid burden but should satisfy at least the WHO/FAO/UNU 2007 safe levels of protein intake [11]. According to data published so far, early diagnosis and treatment in the outcome of organic acidemias are crucial [12,13].

In Poland, IVA was included in the NBS program in 2014. Our centre has years of experience in the assessment and treatment of IVA patients. Within this paper we aim to analyze the long-term outcome of Polish IVA patients.

## 2. Materials and Methods

Ten patients identified between 1989 and 2018 were included in the retrospective analysis. Diagnosis of IVA was made based on tandem MS (increased level of C5 acylcarnitine) and urine GCMS (increased isovalerylglycine and 3-HIVA) results.

Clinical data were collected through the review of patients’ medical records and included age at diagnosis and first symptoms, medical management, and biochemical and clinical outcomes following therapy.

During the follow-up period, each patient has had several ambulatory visits. When metabolic decompensation occurred, patients were admitted to the hospital. Nutritional evaluations and neurological examination, as well as routine laboratory tests such as plasma amino acids and biochemical investigations, were performed every 6–12 months on each visit. All patients had their special diet applied: older children (six patients) had a low-protein, age-appropriate diet, and infants (four patients) had special IVA formulas to provide the proper protein supply. The majority of individuals has followed their diet. Additionally, supplementation was used: glycine and carnitine (in five), glycine (in one patient), carnitine (in four).

To identify molecular defects in the *IVD* gene, Sanger sequencing of coding sequence (exons: 1–11), and exon/intron junction was performed. The nomenclature of identified variants and patients’ genotype follow the Human Genome Variation Society guidelines (HGVS v 2.0, Melbourne, Australia, www.hgvs.org/mutnomen) with referral to the cDNA sequence NM_002225.3 and protein sequence NP_002216.3 of *IVD* gene (*607036; Isovaleryl-CoA dehydrogenase), followed the Human Gene Mutation Database (HGMD, www.hgmd.cf.ac.uk).

Ethical approval (Code 167/KD/2014, date 29 October 2014) of the study protocol was obtained from the Children’s Memorial Health Institute Bioethical Committee, Warsaw, Poland.

## 3. Results

As summarized in Table 1, seven individuals were symptomatic at diagnosis. Five of them were diagnosed at neonatal period and presented the following symptoms: hypotonia (3), failure to thrive (4), vomiting (4), sweaty feet odour (2), and metabolic acidosis (4). Two older children were diagnosed at the age of six years and 10 years when metabolic decompensation due to food intolerance and infection occurred. Two children were diagnosed by family screening (FS), and one child was identified through NBS.

In all patients, a special diet, based on isovaleric acid formula was commenced. Additionally, supplementation was used: glycine and carnitine (25–50 mg/kg/day) in five patients, glycine in one child, and carnitine (30–170 mg/kg/day) in four individuals.

Median follow-up time (median; Q1–Q2) was 2.5 years (1.5–9.0) for NBS and FS children, and 17.0 years (5.0–20.0) for symptomatic patients. Among symptomatic individuals, three (Patients 3, 6, and 9) presented mild neurological symptoms (mild intellectual disability), and one (Patient 2) was severely delayed. He needed gastrostomy tube placement during the decompensation episode, at the age of 4.5 years. After recovery the tube was removed, the patient presents moderate psycho-motor delay (Table 1). Patient 6 was diagnosed with congenital toxoplasmosis, which had no clinical consequences, as hypoacusis or visual problems. Its association with intellectual deficits cannot be proved. This patient has given birth to two children. She underwent a catabolic episode following her first delivery and had to be hospitalized due to that, but both the mother and child did well afterwards. Another symptomatic patient (Patient 5) struggled with depressive disorder and probably due to that his adherence was never satisfactory, which may be the reason for his poor intellectual outcome. The last patient from this group (Patient 9) was in a good clinical state, and except for mild intellectual delay, his neurological issues are normal.

### Molecular Results

Molecular analysis was performed in seven patients leading to detection of biallelic mutations in the *IVD* gene in all of them (Patients 1, 3, 4, 6, 7, 8, and 10). In three others (Patients 2, 5, and 9), genetic diagnosis was not performed. In Patients 7 and 10, no pathogenic SNV (ang. single nucleotide variant) within the coding sequence of the *IDV* gene was identified. The results (no PCR product), however, corresponded to gross deletion. Detailed genotypes of analyzed patients are presented in Table 1.

**Figure 1:**
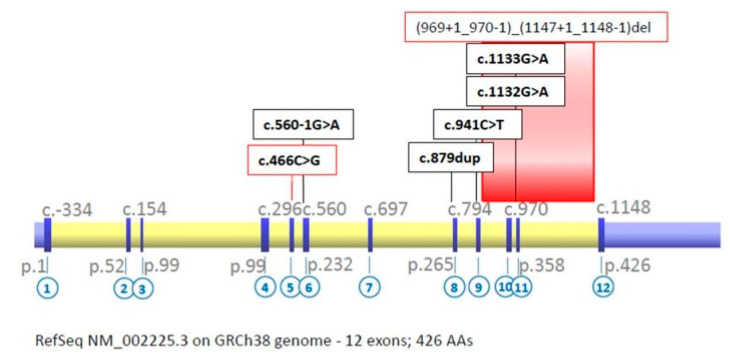
*IVD* gene structure and localization of identified molecular variants (numbers in the circles refer to exons; novel variant are marked in red).

Four of patients were compound heterozygotes and three of them were homozygotes: one for c.1133G>A substitution, and two for gross deletion. In total, seven molecular variants in *IVD* were identified (Table 2). Five of them were known mutations, including four nucleotide changes: missense (c.941C>T, c.1133G>A), splice-site (c.560- 1G>A), frame shift (c.879dup) type and one gross deletion comprising of at least exons 10–11. Additionally, two novel potentially pathogenic variants (c.466C>G, c.1132G>A) were identified (Figure 1). Interestingly, two homozygous patients with deletion on exons 10–11 are not related.

The first missense substitution, c.466C>G, p.(Leu156Val), was noted in three individuals (P3, 4,6) in compound heterozygote state with other known mutations (Table 1). Although there are small physicochemical differences between Leu and Val this variant is localized in highly conserved amino acid of Acyl-CoA dehydrogenase/oxidase, N-terminal domain. Furthermore, the variant is located 1bps upstream of splice acceptor sites, with potentially damaging effect on *IVD* splicing. This change is predicted to be deleterious by in silico analysis using different algorithms (Table 2). The second missense substitution, c.1132G>A, p.(Gly378Ser), was noted only in one patient (P8) in compound heterozygote state with other known mutation as well (Table 1).

The variant is localized in Acyl-CoA dehydrogenase/oxidase C-terminal domain and affects moderately conserved amino acid. Its status is likely pathogenic because known disease-causing substitution was described already in the same amino-acid position (p.Gly378Asp) and a similar, small physicochemical difference occurs between Gly and Ser/Asp, respectively.

## 4. Discussion

Among four classical acidurias (propionic, methylmalonic, glutaric, and isovaleric), IVA seems to have a milder clinical course and the best response to the applied special dietary management. Moreover, the endogenous production of glycine, which is preserved in IVA, additionally neutralizes neurotoxic metabolites, which contributes to a less severe course of the disease. In patients with a mild course of IVA, no negative impact on cognitive development is observed [14].

Our analysis revealed that neurocognitive outcome in individuals with neonatal manifestation and early introduction of treatment was more promising than in patients diagnosed during or after metabolic decompensation [15].

Two of analyzed children were diagnosed at newborn period through FS and by NBS. Therefore, proper intervention began early, and appropriate clinical and neurological outcome has been achieved. With this conclusion, we support the results of study on 21 symptomatic patients with IVA, published in 2012 [16].

Three out of our seven symptomatic patients (43%, Patients 3, 6, and 9) had mild intellectual disability. One boy (Patient 2), who presented with acute neonatal intoxication syndrome at diagnosis, presented the most serious symptoms of the disease. His condition was severe, and due to inability to feed orally, he needed a gastrostomy tube. This does not correspond with the above-mentioned authors’ observation that catabolic episodes do not influence disease course. On the other hand, a girl diagnosed through NBS who has undergone four catabolic episodes due to infections (her adherence is proper) during a five-year follow-up period is doing well, and her development is normal. This observation may suggest that early diagnosis and treatment do not prevent decompensation, but they protect from intellectual impairment. Therefore, it seems that there is another factor that influences the course of the disease, such as IVA accumulation, which is partly dependent on the endogenous production of glycine (Gly).

Five out of ten patients being on medical management present a clinically non-remarkable course of IVA (Patients 1, 4, 6, 8, and 10). Referring to Pinto et al., who emphasized the need for developing treatment recommendations for different groups of IVA patients, i.e., mild vs severe ones, we confirm this necessity with the results presented herein [9]. In our IVA cohort, patients did well on lower doses of both glycine and carnitine comparing to the literature [9]. This suggests that a mild form of the disease is predominant in the Polish population. In addition, since endogenous glycine neutralizes toxic metabolites, a proper diet with leucine restriction may be sufficient in the management of patients with a mild form of IVA. In accordance with Pinto et al., our experience and long-term observation of Polish IVA cohort confirms that attention should be paid to adequate energy intake, not only to protein restriction [9]. Our practice suggests that early diagnosis, either through NBS or FS, resulting in the early introduction of a special diet, might not prevent metabolic decompensations, but provides a good clinical outcome [12,17]. If decompensation occurs, it is milder and without severe, persistent complications. In Poland, since the NBS program was introduced more early forms of IVA has been found, and thus appropriate management could have been commenced at an early stage of the disease. Probably, this has improved IVA course in some cases.

Given the genotype-phenotype correlation, 89 pathogenic variants in the *IVD* gene have been reported so far (HGMD Professional; updated on 4 December 2019). The relationship regarding IVA manifestation is, however, lacking, and the distribution of hotspot varies among populations [13]. In Caucasians, c.941C>T, p.(Ala314Val) missense variant is common, especially in individuals with asymptomatic form [3]. Moreover, some patients with a mild mutation in the *IVD* gene, c.941C>T, p.(Ala314Val), have sufficient endogenous production of this amino acid which conjugates toxic isovaleric acid [6]. They need glycine supplementation only when an excessive number of toxic metabolites from protein catabolism (during stress, illness or fasting) accumulate. Among our patients, this variant was found only in one affected individual, Patient 8, as a compound heterozygote, harbouring a novel variant (c.1132G>A, p.(Gly378Ser)) in the second allele. As his follow-up at the age of 11 years revealed normal psycho-motor development with no decompensation episodes, this variant may result with non-severe IVA phenotype. Moreover, the course of the disease proves the thesis that mildly affected patients need supplementation, especially during catabolic episodes. Concerning the novel variant, c.466C>G, p.(Leu156Val), medical histories of patients in whom it has been identified, suggest that it results in severe IVA course. Two of them (Patients 4 and 6) have been diagnosed based on a positive family history, where the siblings died from IVA in the neonatal period. The third one (Patient 3) was diagnosed based on suggestive symptoms during neonatal period. More cases are however needed to verify our hypothesis.

## 5. Conclusions

All but one of our patients, whose developmental disorders were rather not related to IVA, presented an uncomplicated course of IVA. In early-diagnosed patients, metabolic decompensations, if any, were milder and did not cause severe complications. Generally, the families followed the diet and supplementation, which may be the key point. Obviously, we cannot state what the outcome would be if their adherence was not good, but molecular analyses of the *IVD* gene may help to establish mild IVA forms. Therefore, it is recommended to perform molecular tests which may predict clinical outcomes since specific variants are associated with mild or even asymptomatic IVA courses. Further molecular studies will also allow to confirm the clinical status and genotype–phenotype correlations of novel *IVD* variants reported in our study.

## Figures and Tables

**Table 1:** Clinical and genotype data of 10 Polish patients with isovaleric acidemia (IVA).

	Gender	Age at Diagnosis	Method of Screening (GCMS); -Neonatal -Newborn -Family	First Symptoms	Therapeutic Management	Current Age, Clinical Status and History	*IVD* Genotype *
1	F	neonatal	newborn	adaptation period complicated with congenital infection	special nutritional formula for IVA, Supplementation of Gly and carnitine introduced since diagnosis; good adherence	Age: 5 years normal clinical condition with no complications-4 mild decompensations with no severe complications	c.[1133G>A]; [(1133G>A)] p.(Gly378Asp)/p.(Gly378Asp)
2	M	neonatal	symptomatic	intoxication syndrome: failure to thrive, dehydration, hypotonia ammonia level—761 µmol/l at diagnosis-decompensation	low-protein diet+ special nutrition formula for IVA, Supplementation of Gly and carnitine introduced since diagnosis; satisfactory adherence	Age: 7 years moderate developmental delay, gastrostomy at the age of 4.5 during one of metabolic decompensation, than removed	Na
3	M	neonatal	symptomatic	since birth: hypotonia, apathy, failure to thrive, vomits, specific odour	special nutrition formula for IVA, supplementation of Gly and carnitine introduced since diagnosis; good adherence	Age: 5 years normal clinical condition, average IQ—lower limit of the norm no metabolic decompensations	c.[466C>G(;)879dup] p.(Leu156Val)/p.(Pro294Alafs*38)
4	F	neonatal	Family history positive for IVA (first child died at the age of 8 days due to IVA)	besides light hypotonia no worrying symptoms	special nutrition formula for IVA, supplementation of Gly and carnitine introduced since diagnosis; good adherence	Age: 4 years normal clinical condition and development—2 metabolic decompensations triggered by infections	c.[466C>G(;)1133G> A] p.(Leu156Val)/p.(Gly378Asp)
5	M	neonatal	symptomatic	intoxication syndrome: vomiting, poor general condition—respiratory failure, sweaty foot odour	low-protein diet, supplementation of carnitine introduced since diagnosis; poor adherence	Age: 22 years depression treated with antidepressants, poor adherence to dietary restrictions; 2 metabolic decompensations: first one at diagnosis, second one triggered by infections	Na
6	f	10y	symptomatic	mild mental delay; during 9 and 10 years of age recurrent vomiting and ketosis; inborn toxoplasmosis	low-protein diet, supplementation of carnitine introduced since diagnosis (10 y); poor adherence	Age: 32 years normal clinical condition, gave birth to 2 children -2 decompensations: first at diagnosis, second after 1st childbirth	c.[466C>G(;)560- 1G>A] p.Leu156Val/p.?
7	M	6y	symptomatic	intoxication syndrome: vomiting, encephalopaty, food intolerance	low-Leu diet, supplementation of Gly introduced since diagnosis (6 y); good adherence	Age: 25 years normal psycho-motor development, but recurrent vomiting, multiple decompensatios till diagnosis—since diet introduction—no episodes	c.[(969+1_970-1)_(1147+1_1148-1)del]; [((969+1_970-1)_(1147+1_1148-1)del)] p.?/p.?
8	F	neonatal	symptomatic	good clinical condition	low-protein diet, supplementation of carnitine introduced since diagnosis; good adherence	Age: 11 years normal psycho-motor development—one metabolic decompensation —at diagnosis	c.[941C>T(;)1132G> A] p.(Ala314Val)/p.(Gly378Ser)
9	M	5y	symptomatic	metabolic decompensation: vomiting, poor nutritional status; cerebral palsy	low-protein diet, supplementation of carnitine introduced since diagnosis (5 y); satisfactory adherence	Age: 25 years delayed psycho-motor development—one metabolic decompensation—at diagnosis	Na
10	M	neonatal	family (first child died)	good clinical status	low-protein diet + special formula (Prosobee), supplementation of Gly and carnitine; good adherence	Age: 29 years good clinical, and psychological condition—one decompensation due to bacterial tonsillitis (fever and vomiting)	c.[(969+1_970-1)_(1147+1_1148-1)del]; [((969+1_970-1)_(1147+1_1148-1)del)] p.?/p.?

* The nomenclature of identified genotypes follows the Human Genome Variation Society guidelines (HGVS v 2.0, www.hgvs.org/mutnomen). IVA—isovaleric acidemia; Gly—glicine; Leu—leucine. Low protein diet for IVA = diet with restriction of Gly and Leu. NA—not analyzed/done.

**Table 2:** Additional data on molecular variants identified in *IVD* gene.

*IVD* Variant				Reporting Data *			
cDNA Level	Protein Level	No of Alleles	Type	Status **	HGMD	ClinVar	PIMID
c.466C>G	p.(Leu156Val)	3	missense	novel ^1^	na	Na	no citation
c.560-1G>A	p.?	1	splice-site	known	na	RCV000410323.1 (likely pathogenic-IVA)	no citation
c.879dup	p.(Pro294Alafs * 38)	1	frameshift	known	CI001574 (IVA)	RCV000555769.1 (pathogenic–IV)	10677295
c.941C>T	p.(Ala314Val)	1	missense	known	CM983435 (IVA)	RCV000080003.11(pathogenic–not provided) RCV000003749.9 (pathogenic-IVA)	9665741, 23757202 26018748, 26937393
969+1_970-1)_(1147+1_1148-1)del	p.?	4	deletion (ex.10-11)	known	CP124273 (IVA)	Na	22090376
c.1132G>A	p.(Gly378Ser)	1	missense	Novel ^1^	na	Na	no citation
c.1133G>A	p.(Gly378Asp)	3	missense	known	BM0867669 (IVA?)	Na	19089597

na—not annoted; * Accession number in database ClinVar (https://www.ncbi.nlm.nih.gov/clinvar/), HGMD Professional (http://www.hgmd.cf.ac.uk/ac/index.php) and Pubmed (https://pubmed.ncbi.nlm.nih.gov/?term=IVD) respectively; ** Novel molecular variants were assessed by pathogenicity prediction tools: SIFT, and MutationTaster softwares for missense changes localized in coding sequence and MaxEnt, NNSPLICE or SSF—for nucleotide changes identified in splice-site, respectively. Additionally, the minor allele frequency (MAF) in different public database (e.g., dbSNP, ESP6500, ExAC, gnomAD) were also included in this assessment: ^1^ Highly conserved amino acid (considering 12 species), small physicochemical difference between Leu and Val (Grantham dist.: 32 [0–215]). This variant is localized in Acyl-CoA dehydrogenase/oxidase, N-terminal domain. MAF = 0. In silico prediction result: SIFT (v 6.2.0): Deleterious, MutationTaster (v2013): disease causing. Predicted change at acceptor site 1 bps upstream: + 19.8% (MaxEnt: + 12.5%, NNSPLICE: + 27.2%, SSF: + 6.4%). ^2^ Moderately conserved amino acid (considering 12 species), small physicochemical difference between Gly and Ser (Grantham dist.: 56 [0–215]). This variant is localized in Acyl-CoA dehydrogenase/oxidase C-terminal domain. MAF = 0. In silico prediction result: SIFT: Tolerated, MutationTaster: disease causing.
